# The Interaction between Human Immunodeficiency Virus and Human Papillomaviruses in Heterosexuals in Africa

**DOI:** 10.3390/jcm4040579

**Published:** 2015-04-02

**Authors:** Anna-Lise Williamson

**Affiliations:** Institute of Infectious Disease and Molecular Medicine, Faculty of Health Sciences, University of Cape Town and National Health Laboratory Service, Groote Schuur Hospital, Observatory, Cape Town 7925, South Africa; E-Mail: Anna-Lise.Williamson@uct.ac.za; Tel.: +27-21-406-6124; Fax: +27-21-406-6681

**Keywords:** human papillomavirus, HIV-1, circumcision

## Abstract

Sub-Saharan Africa has the highest incidence of human papillomavirus (HPV) and cervical cancer in the world, which is further aggravated by the burden of human immunodeficiency virus/acquired immunodeficiency syndrome (HIV/AIDS) disease with invasive cervical cancer being an AIDS-defining cancer. The prevalence of HPV infection and associated disease is very high in HIV-infected people and continues to be a problem even after anti-retroviral therapy. In the genital tract, the interaction between HPV and HIV is complex, with infection with multiple HPV types reported to make both women and men more susceptible to HIV infection. Besides the national programmes to vaccinate girls against HPV and screen women for cervical cancer, there should be targeted cervical cancer screening, treatment and prevention programmes introduced into HIV treatment centres. There is evidence that in high HIV prevalence areas, HIV-positive women could cause increases in the prevalence of genital HPV infection in HIV-negative men and so increase the HPV circulating in the community. Condom use and circumcision reduce the acquisition of HIV-1, and also to some extent of HPV. This review will highlight what is known about the interaction of HIV and HPV, with an emphasis on research in Africa.

## 1. Introduction

The International Agency for Research on Cancer has classified both human papillomavirus (HPV) and human immunodeficiency virus type 1 (HIV) as carcinogens: HPV is a direct carcinogen and HIV-1 is an indirect carcinogen through immune suppression. Not all HPV-infected people will develop cancer, but it is important to note that persistent HPV infection, with specific HPV types, and high viral load are important cancer risk predictors [1].

Specific high-risk (HR) types of HPV are causally associated with cervical cancer [2]. Although cervical cancer is a preventable disease, much of the developing world has a high burden of disease due to inadequate cervical cancer screening and treatment programmes [3]. Sub-Saharan Africa has the highest incidence of HPV and cervical cancer in the world [4]. While cervical cancer is the most important cancer, other HPV-associated cancers include head and neck squamous carcinomas, anal, penile, vulvar, vaginal and conjunctival cancer [5,6]. Information these other HPV-associated anogenital cancers in Africa is limited [4]. Moreover, the burden of HPV-associated disease is substantially increased where there is HIV-1 co-infection [7]. HIV-1 and HPVs are both sexually transmitted and are also the cause of significant public health problems in Africa. In the genital tract the interaction between these two viruses is complex, with infection with multiple HR-HPV types reported to make women more susceptible to HIV-1 infection [8,9], while HIV-infected individuals are more likely to have HPV infection [10]. This review will highlight what is known about the interaction of HIV-1 and genital HPV, with an emphasis on research in Africa.

## 2. HPV Infection Increases the Susceptibility to HIV-1 Acquisition

There have now been a number of studies indicating that people infected with genital HPVs are at higher risk of acquiring HIV-1 than people who are not infected with HPV, even after adjusting for risk factors [9,11]. The biological mechanism for this increased susceptibility is not well understood. In a study done on Zimbabwean women incident HIV-1 infection was associated with HPV clearance, indicating that the immune response to HPV may result in an increase of HIV-susceptible cells in the genital tract [12]. Association of HIV-1 acquisition with HPV clearance was also seen in a study on men being circumcised, with increased HIV-1 acquisition correlating with number of HPV genotypes cleared. In this study increased epidermal dendritic cell density was associated with HPV clearance and increased HIV-1 acquisition, which may have increased the number of target cells for HIV-1 infection [13]. CD4+ T cells, the primary infection target of HIV, have been demonstrated to be associated with recent high-grade squamous intraepithelial lesion regression in anal lesions [14], regressing warts [15] and cervical lesion regression [16]. Therefore, the immune response to HPV in mucosal tissue may result in an increase in the number of cells that are susceptible to HIV-1 infection, and accordingly result in an increased incidence of HIV. If HPV infection could be prevented, there could then be an impact on the prevention of HIV-1 acquisition. However in order to impact on HIV-1 acquisition a vaccine that protected against most genital HPV types would be needed, as there are more than 45 HPV types regularly found in the genital tract [17]. There are two effective HPV vaccines available, both protecting against the two highest risk HPV types (HPV 16 and 18), and one also protecting against HPVs 6 and 11, which cause genital warts. The Merck nonavalent HPV vaccine that is currently being tested would be better than both of these, because it protects from infection from 9 HPV types [18,19] but would still not impact on all HPV types.

## 3. The Increase in HPV Infection is an Early Event after HIV-1 Infection

HIV-positive women are known to have a much higher prevalence of HPV than HIV-negative women [20]. Women infected with HIV-1 are more likely to be infected with multiple HPV types soon after HIV-1 infection compared with pre-infection [21,22,23]. Wang *et al.* report that HR HPV infection prevalence in Cape Town women was 20.3% before HIV-1 seroconversion, 23.6% at seroconversion, and 49.1% after seroconversion (*p* = 0.01) [22]. Therefore the increase in HPV prevalence is an early event after HIV-1 infection and is not correlated with a drop in circulating CD4+ cells. Genital warts, which are caused by low-risk (LR) HPV, also increase soon after HIV-1 infection, and it has been speculated that this is due to a higher risk of progression from subclinical or latent infection to clinical HPV disease, rather than to a higher risk of HPV acquisition [24]. In animal models papillomavirus DNA has been demonstrated to persist in the epithelial basal layers, and to be activated after T-cell depletion [25]. Latent HPV could be activated under specific circumstances, including when the immune response is impacted by HIV-1 [26], where T-cell surveillance stops controlling the replication of HPV. A better understanding of the early events influencing HPV control as well as persistence in the genital tract is therefore required. Further research on the impact of HIV on these early events is desirable.

## 4. HIV-1 Infection Increases Prevalence, Persistence and Reduces Clearance of HPV

HPV prevalence is very high in HIV-infected people. In a Cape Town study done on 1371 HIV-positive women and 8050 HIV-negative women aged 17–65 years, the HR-HPV prevalence in cervical samples was higher among HIV-positive women (52.4%) than among HIV-negative women (20.8%) [27]. In the younger age group (17–19 years) 75% of HIV-positive women were HPV-positive compared to 60.2% of HIV-negative women (*p* < 0.06). In the HIV-1 positive *vs.* HIV-negative women aged 25–29 years and 35–39 years, HPV prevalence was 59.7% *vs.* 23.9% (*p* < 0.0001) and 46.4% *vs.* 19.3% (*p* < 0.0001) respectively. Much higher prevalence of HPV was reported in cervical samples from women (median age 31) enrolling at HIV-1 treatment centres in Cape Town, where 78.9% were HPV-positive [28], than the latter study. At another HIV-1 treatment centre in Johannesburg 61% of 1202 cervical samples from HIV-infected women (median age of 38 years) were HR HPV-positive [29]. In a meta-analysis of genital HPV prevalence in African men, prevalence of any HPV was 84.5% (95% CI 74.2 to 91.2) among HIV-positive and 56.4% (95% CI 49.7 to 62.9) among HIV-negative men (*p* < 0.0001) [30]. The natural history of HPV differs in men and women, with women tending to have a higher prevalence of HPV at a young age with a significant drop off in the older age groups. In some populations, older women may have a slight increase in the prevalence of HPV [31]. In HIV-positive women the drop in HPV prevalence is not as dramatic as in HIV-negative women [27]. In men the HPV prevalence tends to be more uniform across age groups regardless of HIV infection [30].

There is evidence that in high HIV-1 prevalence areas, the HPV infected HIV-positive women could result in increases in HPV infection in HIV-negative men [10]. If this is the case then it could be hypothesised that HIV increases the prevalence not only in the HIV-positive population but also within the sexual networks that include HIV-negative partners resulting in increased risk of HPV related disease in this population. In a study done in Cape Town on HIV-1 concordant and discordant heterosexual couples, more HIV-positive men and women had genital HPV compared with HIV-negative men (77 *vs*. 49%; *p* < 0.001) and women (74 *vs*. 36%; *p* < 0.001). Men with HIV-positive partners were at greater risk of HPV infection compared with men with HIV-negative partners. Conversely, the risk of HPV of any type was not found to differ between women with an HIV-positive or HIV-negative male partner. In women, HIV-1 infection and low CD4 count were significantly associated with increased risk of type-specific HPV concordance, but male partner HIV-positive status was not significantly associated with this concordance. Therefore, male genital HPV prevalence and type-specific sharing were influenced by their own HIV-positive status and that of their female partner. In contrast, female genital HPV prevalence and HPV type-specific sharing were determined by their own HIV-positive status but not by that of their male partner [10]. HIV-1 infection was also associated with increased risk of new HPV detection in women (relative risk (RR), 2.98; 95% CI, 2.07–4.29) and men (RR, 2.00; 95% CI, 1.49–2.69). HIV-1 infection reduced the rate of HPV clearance in women (RR, 0.46; 95% CI, 0.34–0.62) and men (RR, 0.71; 95% CI, 0.55–0.93) [32]. Persistent, high viral load, HR-HPV infections are an important risk factor for the development of cancer [33] which is substantially increased in HIV-positive people.

## 5. HPV, HIV-1 and Cervical Cancer

In Africa, as in other parts of the world, the dominant HPV type associated with invasive cervical cancers is HPV-16 [34]. In 515 cancers from Ghana, Nigeria, and South Africa where there was only one HPV type detected, the six most prevalent HPV types were HPV-16 (51.2%), HPV-18 (17.2%), HPV-35 (8.7%), HPV-45 (7.4%), HPV-33 (4.0%) and HPV-52 (2.2%) [35]. HIV-1 positive women have a higher prevalence of multiple HPV type infections in normal, premalignant lesions and invasive cervical cancer (ICC) [27,32,35]. It has been proposed that specific combinations of HPV types increase the risk of ICC and pre-malignant lesions compared to infection with the individual HPV types [36]. High HPV viral loads are associated with increased risk of abnormal cytology [37,38], and HIV-1 co-infection significantly increased the combined alpha-9 taxon HPV viral load in women, but not viral loads for individual HPV types [39].

Invasive cervical cancer is an AIDS-defining cancer: women infected with HIV-1 present with cervical cancer at ages up to 15 years younger than HIV-negative patients [40]. The prevalence of premalignant cervical lesions is increased in HIV-positive women: in a cervical cancer case-control study done in Cape Town, HIV-positive women were nearly 5 times more likely to have HR-HPV infection present compared to HIV-negative women (OR 4.6 (95% CI 2.8–7.5)). Women infected with both HIV-1 and HR-HPV had a more than 40-fold higher risk of squamous intraepithelial lesions than women infected with neither of these viruses [41].

At an HIV-1 treatment clinic in Johannesburg in a cohort of 148 women, 54% had abnormal Papanicolaou smears, with 33% of these assessed as having high grade changes. HPV DNA was found in 95% of the 148 subjects assessed, with 83% having 1 or more HPV oncogenic types. There was a significant risk of an oncogenic HPV type in women with CD4 < 200 cells/μL [20]. Unlike other AIDS defining cancers such as Kaposi’s sarcoma and non-Hodgkin’s lymphoma, the risk of cervical cancer does not decrease after highly active antiretroviral therapy [42]. In addition, even if the women are able to access treatment the recurrence rate is much higher in HIV-positive women, indicating they need close follow up [43,44,45]. The impact of treatment on HPV may depend on the stage of disease. There are conflicting reports on the impact of ARVs on HPV. In one study done in South Africa, anti-retroviral therapy (ART) substantially reduced the detection of HPV where every month on ART significantly reduced the detection risk of any HPV type by 9% which implied increased clearance after initiation of ART [46]. Another study looking at incident HPV detection, failed to show a protective effect of ART in an HIV-positive adolescent cohort [47].

The increased HPV associated disease in HIV-positive individuals has major public health and resource implications for countries with a high HIV-1 prevalence. It is expected that the number of women with cervical cancer is going to increase as more women get access to HIV-1 therapy. There is therefore an urgent need to roll out better cervical screening programmes linked to HIV-1 treatment centres. It is unfortunate that many of the countries in sub-Saharan Africa, with the highest burden of HIV-1 disease, tend to have limited resources to put these programmes in place and to treat premalignant cervical lesions, let alone to treat the cervical cancers [4]. Proposals for cervical screening of HIV positive women include younger age of first screening and more frequent screening protocols with the mode of screening dependent on the resources available [29,48]. Although HPV testing is not as sensitive as visual inspection (5% acetic acid) with digital imaging review or convential cytology in the detection of cervical intraepithelial neoplasia grade 2 and above, the specificity is high [29]. Screen-and-treat regimens using HPV testing reduces high-grade cervical cancer precursors in HIV-infected women.

Anal cancer incidence is greatly increased in HIV-positive individuals, particularly in HIV-positive men who have sex with men (MSM) [49]. The literature on HPV in MSMs is not part of this review and there is very little published on anal HPV and anal cancers in African heterosexuals. Internationally there have been many reports on anal HPV in women particularly in HIV positive women where abnormal anal cytology and HR-HPV prevalences were high [50,51]. Not all anal cancers are associated with HPV [52] but HPV positive patients have a better five-year disease-free survival than HPV-negative patients [53]. HIV-positive women with a history of HPV-associated cervical disease are at increased risk for high-grade anal intraepithelial neoplasia and so screening is recommended. While screening and treatment protocols are not yet well established, Heard *et al.* (2015) demonstrated that anal cytology and HPV-16 genotyping had the best screening performance in their study [51]. There is a need to research anal HPV associated disease and to determine the best screening and treatment protocols for Africa.

## 6. Circumcision Reduces HIV-1 and HPV Acquisition

Male penile circumcision is now regarded as one of the most successful interventions to prevent HIV infection, and many African countries are accordingly offering circumcision on a large scale [54,55]. While women carry the major burden of HPV-associated diseases in the form of cervical cancer, men play a role in transmitting HPV to their partners. Therefore, a reduction of HIV-1 and HPV as a result of circumcision in men could impact significantly on the cervical disease in their communities. In randomised controlled trials of voluntary surgical male circumcision *versus* no circumcision in HIV-negative men in sub-Saharan Africa, the incidence of HIV-1 was reduced between 38% and 66% over 24 months [56]. Male circumcision reduces the acquisition and persistence of high HPV viral load infections in the glans in men [57] as well as decreases the HR HPV viral load in female partners [58]. In addition since HPV infection may increase the risk of HIV-1 acquisition [11], a reduction in HPV may also help reduce the acquisition of HIV-1 [59].

## 7. Impact of Microbicides on HIV-1 and HPV

Vaginal microbicides are actively being tested for the prevention of HIV-1 infections. It is desirable, however, that microbicides also prevent other sexually transmitted infections. Only one microbicide has been demonstrated to prevent HIV: this is a tenofovir-containing microbicide gel [60]. The impact of this microbicide on HPV has not yet been tested. In a trial of the nonoxynol-9-based vaginal gel COL-1492 in sex workers there was an increase in HIV-1 in the women using the gel compared to non-users, indicating that the gel may have enhanced HIV-1 acquisition [61]. A significant increase in multiple HPV infections in HIV-1 seronegative women using nonoxynol-9 compared with HIV-1 seronegative women using placebo (OR 3.5 95% CI 1.0–11.8) was also observed in the South African arm of the trial, demonstrating that the use of nonoxynol-9 did not prevent genital HPV infection and could increase the virus’ ability to infect or persist [62]. In contrast, while a randomised double-blind placebo-controlled trial found that the carrageenan-based vaginal microbicide Carraguard was unable to prevent HIV-1 infection [63], there was some efficacy in preventing HPV infection. In this trial there were 348 compliant women (174 Carraguard, 174 placebo users) with relatively high adherence to gel use, who inserted 80% of their opened, returned applicators of test product with the proportion of applicator insertions to sex acts >30%. After adjusting for risk factors, these compliant Carraguard users were 0.62 as likely to be classified HR-HPV positive (95% CI 0.41–0.94) as compliant placebo users. This is the first report showing a negative association of HPV infection with a vaginal microbicide [64]. This was confirmed in a mouse model where carrageenan was shown to protect from HPV infection, while nonoxynol-9 increased HPV infection [65]. There is therefore a need for a randomised control trial to confirm that carrageenan based microbicides reduce HPV infection. There are microbicide gels in preclinical development. One promising gel, MZC, containing MIV-150, zinc acetate and carrageenan protected macaques against simian-human immunodeficiency virus significantly reduced vaginal and anorectal HSV-2 infection of mice as well as HPV16 pseudovirus infection [66].

## 8. Condoms, HIV-1 and HPV

Consistent use of condoms has the potential to reduce both HIV-1 and HPV acquisition. In a multinational HPV cohort study the risk of HPV acquisition was 2-fold lower among men with no steady sex partner who always used condoms, compared with those who never used condoms (hazard ratio, 0.54), after adjustment for country, age, race, education duration, smoking, alcohol, and number of recent sex partners [67]. However, condoms are more effective against HIV-1 than HPV due to the comparatively larger area available for HPV infection and transmission in the male and female genital tract, compared to HIV. Condoms are effective at preventing HIV-1 transmission with the level of protection approximating 87% [68].

## 9. HPV Vaccines in HIV-1 Positive Women

While there have been no efficacy studies done on the available HPV vaccines in HIV-1 positive individuals, there have been a number of safety and immunogenicity studies. A study of the quadrivalent Merck HPV vaccine in HIV-infected and HIV-negative adolescents and young adults aged 13–27 years showed that the vaccine was generally safe and well tolerated, with the rate of seroconversion being 85% in HIV-infected and 91% in HIV-negative subjects, with antibody titres being slightly lower in the HIV-positive children [69]. In another trial of the quadrivalent HPV vaccine on 319 HIV-infected women (median age 36 years) in the United States, Brazil, and South Africa there were no safety issues identified. The seroconversion proportions for HPV types 6, 11, 16, and 18 in women with CD4+ T-cell counts above 350 cells/μL were 96%, 98%, 99%, and 91% respectively, while those women with CD4+ counts less than <200 cells/μL had conversion proportions of 84%, 92%, 93%, and 75%, respectively [70]. These results show that even in women with AIDS it is possible to elicit a relatively good immune response to the quadrivalent HPV vaccine. The bivalent Glaxosmithkline (GSK) bivalent HPV vaccine was found to be safe in asymptomatic HIV-positive women aged 18–25 years. Irrespective of baseline HPV status women, who received the HPV-16/18 vaccine were seropositive for both HPV-16 and HPV-18 after the second vaccine dose (month 2), and remained seropositive for both antigens at month 12. The vaccine did not have any impact on CD4^+^ T-cell count, HIV viral load or HIV clinical stage [71]. These studies prepare the way for routine HPV vaccination of HIV-1 infected women. While most national HPV vaccine programmes target adolescents before they are sexually active, there are also catch-up programmes that target older girls [72]. There are also many examples of reduction in HPV-associated disease after vaccination in HIV-negative populations [73,74]. Ideally, one would like to vaccinate HIV-1 positive women even after they have been exposed to HPV—however, it remains to be shown whether vaccination of these women will indeed reduce the incidence of cervical disease. Nonetheless, the high burden of HIV-1 and HPV disease in Africa justifies the widespread introduction of HPV vaccination [75]. Efficacy trials are needed to formulate policy on vaccination of HIV-positive individuals.

## 10. Conclusions

In recent years there has been a substantial increase in the number of people receiving ART to treat HIV-1 infections, and so mortality has been significantly reduced [76]. HIV-positive women have higher incidence and quicker progression of cervical neoplasia [77]; thus, as more women receive treatment for HIV-1, the HPV-associated cancers are expected to increase in populations where HIV-1 is endemic. Countries in sub-Saharan Africa are particularly hard hit. An integrated response to prevent these cancers is urgently required, with cervical cancer prevention programmes being integrated into HIV-1 and tuberculosis treatment centres. HPV vaccination of young girls should be introduced, and cervical cancer screening and treatment programmes should be improved.

## References

[1] Chen C.J., Hsu W.L., Yang H.I., Lee M.H., Chen H.C., Chien Y.C., You S.L. (2014). Epidemiology of virus infection and human cancer. Recent Results Cancer Res..

[2] Zur Hausen H. (1991). Human papillomaviruses in the pathogenesis of anogenital cancer. Virology.

[3] Denny L. (2012). Cervical cancer: Prevention and treatment. Discov. Med..

[4] De Vuyst H., Alemany L., Lacey C., Chibwesha C.J., Sahasrabuddhe V., Banura C., Denny L., Parham G.P. (2013). The burden of human papillomavirus infections and related diseases in sub-Saharan Africa. Vaccine.

[5] Zur Hausen H. (2009). Papillomaviruses in the causation of human cancers—A brief historical account. Virology.

[6] Mwololo A., Nyagol J., Rogena E., Ochuk W., Kimani M., Onyango N., Pacenti L., Santopietro R., Leoncini L., Mwanda W. (2014). Correlation of EGFR, pEGFR and p16INK4 expressions and high risk HPV infection in HIV/AIDS-related squamous cell carcinoma of conjunctiva. Infect. Agents Cancer.

[7] Pantanowitz L., Michelow P. (2011). Review of human immunodeficiency virus (HIV) and squamous lesions of the uterine cervix. Diagn. Cytopathol..

[8] Auvert B., Marais D., Lissouba P., Zarca K., Ramjee G., Williamson A.L. (2011). High-risk human papillomavirus is associated with HIV acquisition among South African female sex workers. Infect. Dis. Obstet. Gynecol..

[9] Houlihan C.F., Larke N.L., Watson-Jones D., Smith-McCune K.K., Shiboski S., Gravitt P.E., Smith J.S., Kuhn L., Wang C., Hayes R. (2012). Human papillomavirus infection and increased risk of HIV acquisition. A systematic review and meta-analysis. Aids.

[10] Mbulawa Z.Z., Marais D.J., Johnson L.F., Boulle A., Coetzee D., Williamson A.L. (2010). Influence of human immunodeficiency virus and CD4 count on the prevalence of human papillomavirus in heterosexual couples. J. Gen. Virol..

[11] Lissouba P., Van de Perre P., Auvert B. (2013). Association of genital human papillomavirus infection with HIV acquisition: A systematic review and meta-analysis. Sex. Transm. Infect..

[12] Averbach S.H., Gravitt P.E., Nowak R.G., Celentano D.D., Dunbar M.S., Morrison C.S., Grimes B., Padian N.S. (2010). The association between cervical human papillomavirus infection and HIV acquisition among women in Zimbabwe. Aids.

[13] Tobian A.A., Grabowski M.K., Kigozi G., Redd A.D., Eaton K.P., Serwadda D., Cornish T.C., Nalugoda F., Watya S., Buwembo D. (2013). Human papillomavirus clearance among males is associated with HIV acquisition and increased dendritic cell density in the foreskin. J. Infect. Dis..

[14] Tong W.W., Shepherd K., Garland S., Meagher A., Templeton D.J., Fairley C.K., Jin F., Poynten I.M., Zaunders J., Hillman R.J. (2015). Human papillomavirus 16-specific T-cell responses and spontaneous regression of anal high-grade squamous intraepithelial lesions. J. Infect. Dis..

[15] Coleman N., Birley H.D., Renton A.M., Hanna N.F., Ryait B.K., Byrne M., Taylor-Robinson D., Stanley M.A. (1994). Immunological events in regressing genital warts. Am. J. Clin. Pathol..

[16] Kim K.H., Greenfield W.W., Cannon M.J., Coleman H.N., Spencer H.J., Nakagawa M. (2012). CD4+ T-cell response against human papillomavirus type 16 E6 protein is associated with a favorable clinical trend. Cancer Immunol. Immunother. CII.

[17] Ameur A., Meiring T.L., Bunikis I., Haggqvist S., Lindau C., Lindberg J.H., Gustavsson I., Mbulawa Z.Z., Williamson A.L., Gyllensten U. (2014). Comprehensive profiling of the vaginal microbiome in HIV positive women using massive parallel semiconductor sequencing. Sci. Rep..

[18] Van de Velde N., Boily M.C., Drolet M., Franco E.L., Mayrand M.H., Kliewer E.V., Coutlee F., Laprise J.F., Malagon T., Brisson M. (2012). Population-level impact of the bivalent, quadrivalent, and nonavalent human papillomavirus vaccines: A model-based analysis. J. Natl. Cancer Inst..

[19] Joura E.A., Giuliano A.R., Iversen O.E., Bouchard C., Mao C., Mehlsen J., Moreira E.D., Ngan Y., Petersen L.K., Lazcano-Ponce E. (2015). A 9-valent HPV vaccine against infection and intraepithelial neoplasia in women. N. Engl. J. Med..

[20] Firnhaber C., Zungu K., Levin S., Michelow P., Montaner L.J., McPhail P., Williamson A.L., Allan B.R., Van der Horst C., Rinas A. (2009). Diverse and high prevalence of human papillomavirus associated with a significant high rate of cervical dysplasia in human immunodeficiency virus-infected women in Johannesburg, South Africa. Acta Cytol..

[21] Marais D.J., Carrara H., Ramjee G., Kay P., Williamson A.L. (2009). HIV-1 seroconversion promotes rapid changes in cervical human papillomavirus (HPV) prevalence and HPV-16 antibodies in female sex workers. J. Med. Virol..

[22] Wang C., Wright T.C., Denny L., Kuhn L. (2011). Rapid rise in detection of human papillomavirus (HPV) infection soon after incident HIV infection among South African women. J. Infect. Dis..

[23] Nowak R.G., Gravitt P.E., Morrison C.S., Gange S.J., Kwok C., Oliver A.E., Howard R., Van der Pol B., Salata R.A., Padian N.S. (2011). Increases in human papillomavirus detection during early HIV infection among women in Zimbabwe. J. Infect. Dis..

[24] Chirgwin K.D., Feldman J., Augenbraun M., Landesman S., Minkoff H. (1995). Incidence of venereal warts in human immunodeficiency virus-infected and uninfected women. J. Infect. Dis..

[25] Maglennon G.A., McIntosh P.B., Doorbar J. (2014). Immunosuppression facilitates the reactivation of latent papillomavirus infections. J. Virol..

[26] Doorbar J. (2013). Latent papillomavirus infections and their regulation. Curr. Opin. Virol..

[27] McDonald A.C., Tergas A.I., Kuhn L., Denny L., Wright T.C. (2014). Distribution of human papillomavirus genotypes among HIV-positive and HIV-negative women in Cape Town, South Africa. Front. Oncol..

[28] Moodley J.R., Constant D., Hoffman M., Salimo A., Allan B., Rybicki E., Hitzeroth I., Williamson A.L. (2009). Human papillomavirus prevalence, viral load and pre-cancerous lesions of the cervix in women initiating highly active antiretroviral therapy in South Africa: A cross-sectional study. BMC Cancer.

[29] Firnhaber C., Mayisela N., Mao L., Williams S., Swarts A., Faesen M., Levin S., Michelow P., Omar T., Hudgens M.G. (2013). Validation of cervical cancer screening methods in HIV positive women from Johannesburg South Africa. PLoS ONE.

[30] Olesen T.B., Munk C., Christensen J., Andersen K.K., Kjaer S.K. (2014). Human papillomavirus prevalence among men in sub-Saharan Africa: A systematic review and meta-analysis. Sex. Transm. Infect..

[31] De Sanjose S., Diaz M., Castellsague X., Clifford G., Bruni L., Munoz N., Bosch F.X. (2007). Worldwide prevalence and genotype distribution of cervical human papillomavirus DNA in women with normal cytology: A meta-analysis. Lancet Infect. Dis..

[32] Mbulawa Z.Z., Marais D.J., Johnson L.F., Coetzee D., Williamson A.L. (2012). Impact of human immunodeficiency virus on the natural history of human papillomavirus genital infection in South African men and women. J. Infect. Dis..

[33] Rositch A.F., Koshiol J., Hudgens M.G., Razzaghi H., Backes D.M., Pimenta J.M., Franco E.L., Poole C., Smith J.S. (2013). Patterns of persistent genital human papillomavirus infection among women worldwide: A literature review and meta-analysis. Int. J. Cancer J. Int. Cancer.

[34] Guan P., Howell-Jones R., Li N., Bruni L., de Sanjose S., Franceschi S., Clifford G.M. (2012). Human papillomavirus types in 115,789 HPV-positive women: A meta-analysis from cervical infection to cancer. Int. J. Cancer J. Int. Cancer.

[35] Denny L., Adewole I., Anorlu R., Dreyer G., Moodley M., Smith T., Snyman L., Wiredu E., Molijn A., Quint W. (2014). Human papillomavirus prevalence and type distribution in invasive cervical cancer in sub-Saharan Africa. Int. J. Cancer J. Int. Cancer.

[36] Carrillo-Garcia A., Ponce-de-Leon-Rosales S., Cantu-de-Leon D., Fragoso-Ontiveros V., Martinez-Ramirez I., Orozco-Colin A., Mohar A., Lizano M. (2014). Impact of human papillomavirus coinfections on the risk of high-grade squamous intraepithelial lesion and cervical cancer. Gynecol. Oncol..

[37] Wang S.M., Colombara D., Shi J.F., Zhao F.H., Li J., Chen F., Chen W., Li S.M., Zhang X., Pan Q.J. (2013). Six-year regression and progression of cervical lesions of different human papillomavirus viral loads in varied histological diagnoses. Int. J. Gynecol. Cancer.

[38] Depuydt C.E., Criel A.M., Benoy I.H., Arbyn M., Vereecken A.J., Bogers J.J. (2012). Changes in type-specific human papillomavirus load predict progression to cervical cancer. J. Cell. Mol. Med..

[39] Mbulawa Z.Z., Johnson L.F., Marais D.J., Gustavsson I., Moodley J.R., Coetzee D., Gyllensten U., Williamson A.L. (2014). Increased alpha-9 human papillomavirus species viral load in human immunodeficiency virus positive women. BMC Infect. Dis..

[40] Moodley M., Moodley J., Kleinschmidt I. (2001). Invasive cervical cancer and human immunodeficiency virus (HIV) infection: A South African perspective. Int. J. Gynecol. Cancer.

[41] Moodley J.R., Hoffman M., Carrara H., Allan B.R., Cooper D.D., Rosenberg L., Denny L.E., Shapiro S., Williamson A.L. (2006). HIV and pre-neoplastic and neoplastic lesions of the cervix in South Africa: A case-control study. BMC Cancer.

[42] Cobucci R.N., Lima P.H., de Souza P.C., Costa V.V., Cornetta M.D., Fernandes J.V., Goncalves A.K. (2014). Assessing the impact of HAART on the incidence of defining and non-defining AIDS cancers among patients with HIV/AIDS: A systematic review. J. Infect. Public Health.

[43] Russomano F., Paz B.R., Camargo M.J., Grinstejn B.G., Friedman R.K., Tristao M.A., Oliveira C.A. (2013). Recurrence of cervical intraepithelial neoplasia in human immunodeficiency virus-infected women treated by means of electrosurgical excision of the transformation zone (LLETZ) in Rio de Janeiro, Brazil. Sao Paulo Med. J..

[44] Mungo C., Cohen C.R., Maloba M., Bukusi E.A., Huchko M.J. (2013). Prevalence, characteristics, and outcomes of HIV-positive women diagnosed with invasive cancer of the cervix in Kenya. Int. J. Gynaecol. Obstet..

[45] Foulot H., Heard I., Potard V., Costagliola D., Chapron C. (2008). Surgical management of cervical intraepithelial neoplasia in HIV-infected women. Eur. J. Obstet. Gynecol. Reprod. Biol..

[46] Zeier M.D., Botha M.H., Engelbrecht S., Machekano R.N., Jacobs G.B., Isaacs S., van Schalkwyk M., van der Merwe H., Mason D., Nachega J.B. (2015). Combination antiretroviral therapy reduces the detection risk of cervical human papilloma virus infection in women living with HIV. Aids.

[47] Shrestha S., Sudenga S.L., Smith J.S., Bachmann L.H., Wilson C.M., Kempf M.C. (2010). The impact of highly active antiretroviral therapy on prevalence and incidence of cervical human papillomavirus infections in HIV-positive adolescents. BMC Infect. Dis..

[48] Kuhn L., Wang C., Tsai W.Y., Wright T.C., Denny L. (2010). Efficacy of human papillomavirus-based screen-and-treat for cervical cancer prevention among HIV-infected women. Aids.

[49] Denny L.A., Franceschi S., de Sanjose S., Heard I., Moscicki A.B., Palefsky J. (2012). Human papillomavirus, human immunodeficiency virus and immunosuppression. Vaccine.

[50] Cambou M.C., Luz P.M., Lake J.E., Levi J.E., Coutinho J.R., de Andrade A., Heinke T., Derrico M., Veloso V.G., Friedman R.K. (2015). Anal human papillomavirus (HPV) prevalences and factors associated with abnormal anal cytology in HIV-infected women in an urban cohort from Rio de Janeiro, Brazil. AIDS Patient Care STDs.

[51] Heard I., Etienney I., Potard V., Poizot-Martin I., Moore C., Lesage A.C., Ressiot E., Crenn-Hebert C., Flejou J.F., Cubie H. (2015). High prevalence of anal human papillomavirus-associated cancer precursors in a contemporary cohort of asymptomatic HIV-infected women. Clin. Infect. Dis..

[52] Alemany L., Saunier M., Alvarado-Cabrero I., Quiros B., Salmeron J., Shin H.R., Pirog E.C., Guimera N., Hernandez-Suarez G., Felix A. (2015). Human papillomavirus DNA prevalence and type distribution in anal carcinomas worldwide. Int. J. Cancer.

[53] Ravenda P.S., Magni E., Botteri E., Manzotti M., Barberis M., Vacirca D., Trovato C.M., Dell’Acqua V., Leonardi M.C., Sideri M. (2014). Prognostic value of human papillomavirus in anal squamous cell carcinoma. Cancer Chemother. Pharmacol..

[54] Auvert B., Taljaard D., Rech D., Lissouba P., Singh B., Bouscaillou J., Peytavin G., Mahiane S.G., Sitta R., Puren A. (2013). Association of the ANRS-12126 male circumcision project with HIV levels among men in a South African township: Evaluation of effectiveness using cross-sectional surveys. PLoS Med..

[55] Centers for Disease Control and Prevention (CDC) (2013). Voluntary medical male circumcision—Southern and eastern Africa, 2010–2012. MMWR Morb. Mortal. Wkly. Rep..

[56] Siegfried N., Muller M., Deeks J.J., Volmink J. (2009). Male circumcision for prevention of heterosexual acquisition of HIV in men. Cochrane Database Syst. Rev..

[57] Senkomago V., Backes D.M., Hudgens M.G., Poole C., Agot K., Moses S., Snijders P.J., Meijer C.J., Hesselink A.T., Schlecht N.F. (2015). Acquisition and persistence of high viral load HPV16 and HPV18 infections in men enrolled in a circumcision trial in Kisumu, Kenya. J. Infect. Dis..

[58] Davis M.A., Gray R.H., Grabowski M.K., Serwadda D., Kigozi G., Gravitt P.E., Nalugoda F., Watya S., Wawer M.J., Quinn T.C. (2013). Male circumcision decreases high-risk human papillomavirus viral load in female partners: A randomized trial in Rakai, Uganda. Int. J. Cancer.

[59] Rositch A.F., Mao L., Hudgens M.G., Moses S., Agot K., Backes D.M., Nyagaya E., Snijders P.J., Meijer C.J., Bailey R.C. (2014). Risk of HIV acquisition among circumcised and uncircumcised young men with penile human papillomavirus infection. Aids.

[60] Abdool Karim Q., Abdool Karim S.S., Frohlich J.A., Grobler A.C., Baxter C., Mansoor L.E., Kharsany A.B., Sibeko S., Mlisana K.P., Omar Z. (2010). Effectiveness and safety of tenofovir gel, an antiretroviral microbicide, for the prevention of HIV infection in women. Science.

[61] Van Damme L., Ramjee G., Alary M., Vuylsteke B., Chandeying V., Rees H., Sirivongrangson P., Mukenge-Tshibaka L., Ettiegne-Traore V., Uaheowitchai C. (2002). Effectiveness of COL-1492, a nonoxynol-9 vaginal gel, on HIV-1 transmission in female sex workers: A randomised controlled trial. Lancet.

[62] Marais D., Carrara H., Kay P., Ramjee G., Allan B., Williamson A.L. (2006). The impact of the use of COL-1492, a nonoxynol-9 vaginal gel, on the presence of cervical human papillomavirus in female sex workers. Virus Res..

[63] Ramjee G., Kamali A., McCormack S. (2010). The last decade of microbicide clinical trials in Africa: From hypothesis to facts. Aids.

[64] Marais D., Gawarecki D., Allan B., Ahmed K., Altini L., Cassim N., Gopolang F., Hoffman M., Ramjee G., Williamson A.L. (2011). The effectiveness of Carraguard, a vaginal microbicide, in protecting women against high-risk human papillomavirus infection. Antivir. Ther..

[65] Roberts J.N., Buck C.B., Thompson C.D., Kines R., Bernardo M., Choyke P.L., Lowy D.R., Schiller J.T. (2007). Genital transmission of HPV in a mouse model is potentiated by nonoxynol-9 and inhibited by carrageenan. Nat. Med..

[66] Kizima L., Rodriguez A., Kenney J., Derby N., Mizenina O., Menon R., Seidor S., Zhang S., Levendosky K., Jean-Pierre N. (2014). A potent combination microbicide that targets SHIV-RT, HSV-2 and HPV. PLoS ONE.

[67] Pierce Campbell C.M., Lin H.Y., Fulp W., Papenfuss M.R., Salmeron J.J., Quiterio M.M., Lazcano-Ponce E., Villa L.L., Giuliano A.R. (2013). Consistent condom use reduces the genital human papillomavirus burden among high-risk men: The HPV infection in men study. J. Infect. Dis..

[68] Davis K.R., Weller S.C. (1999). The effectiveness of condoms in reducing heterosexual transmission of HIV. Fam. Plan. Perspect..

[69] Giacomet V., Penagini F., Trabattoni D., Vigano A., Rainone V., Bernazzani G., Bonardi C.M., Clerici M., Bedogni G., Zuccotti G.V. (2014). Safety and immunogenicity of a quadrivalent human papillomavirus vaccine in HIV-infected and HIV-negative adolescents and young adults. Vaccine.

[70] Kojic E.M., Kang M., Cespedes M.S., Umbleja T., Godfrey C., Allen R.T., Firnhaber C., Grinsztejn B., Palefsky J.M., Webster-Cyriaque J.Y. (2014). Immunogenicity and safety of the quadrivalent human papillomavirus vaccine in HIV-1-infected women. Clin. Infect. Dis..

[71] Denny L., Hendricks B., Gordon C., Thomas F., Hezareh M., Dobbelaere K., Durand C., Herve C., Descamps D. (2013). Safety and immunogenicity of the HPV-16/18 AS04-adjuvanted vaccine in HIV-positive women in South Africa: A partially-blind randomised placebo-controlled study. Vaccine.

[72] Burger E.A., Sy S., Nygard M., Kristiansen I.S., Kim J.J. (2015). Too late to vaccinate? The incremental benefits and cost-effectiveness of a delayed catch-up program using the 4-valent human papillomavirus vaccine in Norway. J. Infect. Dis..

[73] Chow E.P., Read T.R., Wigan R., Donovan B., Chen M.Y., Bradshaw C.S., Fairley C.K. (2014). Ongoing decline in genital warts among young heterosexuals 7 years after the Australian human papillomavirus (HPV) vaccination programme. Sex. Transm. Infect..

[74] Pollock K.G., Kavanagh K., Potts A., Love J., Cuschieri K., Cubie H., Robertson C., Cruickshank M., Palmer T.J., Nicoll S. (2014). Reduction of low- and high-grade cervical abnormalities associated with high uptake of the HPV bivalent vaccine in Scotland. Br. J. Cancer.

[75] Kim J.J., Campos N.G., O’Shea M., Diaz M., Mutyaba I. (2013). Model-based impact and cost-effectiveness of cervical cancer prevention in sub-Saharan Africa. Vaccine.

[76] Mutevedzi P.C., Newell M.L. (2014). The changing face of the HIV epidemic in sub-Saharan Africa. Trop. Med. Int. Health TM IH.

[77] Denslow S.A., Rositch A.F., Firnhaber C., Ting J., Smith J.S. (2014). Incidence and progression of cervical lesions in women with HIV: A systematic global review. Int. J. STD AIDS.

